# Supplementary material for: Complexome profiling of the Chlamydomonas psb28 mutant reveals TEF5 as an early PSII assembly factor
Source: Plant Cell. 2025 Mar 17;37(6):koaf055. doi: 10.1093/plcell/koaf055 (PMC12164590; doi:10.1093/plcell/koaf055)
Supplement: koaf055_Supplementary_Data [file koaf055_supplementary_data.zip › psb28_interactive.zip!/plots/intensity/qualityPlots/peptides/ASA1_Cre07.g340350.t1.1.html]

### Cre07.g340350.t1.1

Trivial name: ASA1  
SeqCoverage [median]: 54.10  
Mass: 63.16 kDa  
  
 

| Sequence | MissedCleavage | Mass [Da] | wtIntensity | muIntensity | Score |
| --- | --- | --- | --- | --- | --- |
| AELKPVYGDSVDVAK | 1 | 1589.83 | 115904.13 | 129149.92 | 210.02 |
| AEQEAADNAYLK | 0 | 1321.62 | 41826.77 | 69830.29 | 272.94 |
| AQNLKPGEVGK | 1 | 1139.63 | 132340.44 | 174440.25 | 163.83 |
| DLGDSKGEPLLK | 1 | 1270.68 | 17903.35 | 47905.23 | 112.50 |
| DVDGQLASK | 0 | 931.46 | 2880.85 | 4572.33 | 100.02 |
| EATEGLLK | 0 | 859.47 | 72105.52 | 109757.41 | 117.16 |
| ESTALLDEWK | 0 | 1190.58 | 9553.53 | 20120.13 | 128.36 |
| FELLAPQYAELLQAASSGSK | 0 | 2122.09 | 7041.30 | 0.00 | 87.52 |
| FFDTVLAK | 0 | 939.51 | 129274.18 | 255074.25 | 200.12 |
| FFDTVLAKR | 1 | 1095.61 | 3305.69 | 15344.18 | 98.03 |
| HFGVDLPHSVIAHMDPVLIK | 0 | 2224.18 | 10389.78 | 56117.68 | 166.91 |
| HFGVDLPHSVIAHMDPVLIKK | 1 | 2352.28 | 0.00 | 6828.97 | 66.78 |
| KEATEGLLK | 1 | 987.56 | 4753.20 | 0.00 | 120.99 |
| KLSAGDQELVSK | 1 | 1273.69 | 29579.17 | 41661.04 | 187.11 |
| KQVGHFEPELVAGR | 1 | 1565.83 | 39935.40 | 62239.98 | 143.76 |
| KSEAEAAAASK | 1 | 1061.54 | 50021.58 | 77642.98 | 253.10 |
| KVLVSPEVAAVPLK | 1 | 1448.90 | 1575.91 | 6698.92 | 66.53 |
| KVLVSPEVAAVPLKDVDGQLASK | 2 | 2362.35 | 11036.18 | 32638.58 | 128.98 |
| LLQSYK | 0 | 750.43 | 24228.92 | 54567.54 | 134.12 |
| LLQSYKDLGDSK | 1 | 1365.71 | 6665.03 | 13759.03 | 132.14 |
| LLQSYKDLGDSKGEPLLK | 2 | 2003.09 | 27906.10 | 110498.29 | 105.39 |
| LSAGDQELVSK | 0 | 1145.59 | 124233.17 | 148249.62 | 280.25 |
| MSHHPALDSFLLK | 0 | 1494.77 | 1996.10 | 13828.18 | 130.10 |
| MSHHPALDSFLLKR | 1 | 1650.87 | 9485.06 | 77246.26 | 165.51 |
| NKESTALLDEWK | 1 | 1432.72 | 9043.49 | 36448.18 | 90.79 |
| PVSLDAVNK | 0 | 941.52 | 0.00 | 2980.96 | 93.47 |
| QKELAAR | 1 | 814.47 | 0.00 | 1638.26 | 170.87 |
| QVGHFEPELVAGR | 0 | 1437.74 | 50936.47 | 81464.20 | 164.53 |
| SAEFRAEQEAADNAYLK | 1 | 1911.90 | 34194.57 | 58881.51 | 170.11 |
| SEAEAAAASK | 0 | 933.44 | 15179.44 | 12990.36 | 191.45 |
| SFEDLTAPVPNFR | 0 | 1491.74 | 104833.75 | 119023.30 | 264.90 |
| SKKEATEGLLK | 2 | 1202.69 | 3988.64 | 7532.46 | 110.88 |
| SLEPAKK | 1 | 771.45 | 20364.22 | 31707.36 | 134.85 |
| TPAQQIADR | 0 | 998.51 | 130490.59 | 116400.65 | 227.08 |
| TPAQQIADRQK | 1 | 1254.67 | 2301.59 | 9186.79 | 143.22 |
| TPAQQIADRQKELAAR | 2 | 1794.97 | 0.00 | 3336.67 | 124.78 |
| VEFSEGVAAPK | 0 | 1132.58 | 29939.41 | 44585.21 | 178.94 |
| VLVSPEVAAVPLK | 0 | 1320.80 | 7656.16 | 9137.71 | 118.40 |
| VLVSPEVAAVPLKDVDGQLASK | 1 | 2234.25 | 56372.02 | 126829.14 | 127.32 |
| VTDAYLK | 0 | 808.43 | 45494.57 | 81174.43 | 143.02 |
| YAVTKK | 1 | 708.42 | 7641.08 | 7479.54 | 134.12 |
| YKAEEDALR | 1 | 1093.54 | 52214.22 | 72038.98 | 185.61 |
| YKAEEDALRAELKPVYGDSVDVAK | 3 | 2665.36 | 6071.37 | 47906.35 | 105.32 |
| YSADRYLYK | 1 | 1177.58 | 13381.21 | 8937.55 | 76.94 |
|  |  |  | 1464044.1 | 2407850.7 |  |
